# The effect of maternal sodium ρ-perfluorous nonenoxybenzene sulfonate exposure on the gut microbiota in dams and offspring

**DOI:** 10.1038/s41598-025-15021-9

**Published:** 2025-08-25

**Authors:** Caiyun Wang, Feifei Ping, Qian Tong, Yanyan Li, Yuanxiang Jin

**Affiliations:** 1 School of Public Health, Changzhi Medical College, 161, Jiefang East Street, Changzhi, 046000 Shanxi China; 2 The Key Laboratory of Environmental Pathogenic Mechanisms and Prevention of Chronic Diseases, Changzhi Medical College, Changzhi, China; 3 Laboratory of Environmental Factors and Population Health, Beijing, China; 4 Department of Occupational Health, Changzhi Disease Prevention and Control Center, Changzhi, 046000 Shanxi China; 5 College of Biotechnology and Bioengineering, Zhejiang University of Technology, 18, Chaowang Road, Hangzhou, 310032 Zhejiang Province China

**Keywords:** Sodium ρ-perfluorous nonenoxybenzene sulfonate (OBS), Gut microbiota, Metabolic pathways, Offspring, Environmental chemistry, Environmental impact

## Abstract

Sodium ρ-perfluorous nonenoxybenzene sulfonate (OBS) is increasingly used as an effective perfluoroalkyl/polyfluoroalkyl substances (PFASs) alternative across multiple industries. This study involves exposing pregnant C57BL/6 mice to OBS at concentrations of 0, 0.5, and 5.0 mg/L via drinking water during gestation and lactation. The investigation focused on analyzing gut microbiota in both dams and offspring after maternal OBS exposure. Results highlighted notable changes in the gut microbiota composition within the colonic content of both dams and offspring, the *Bacteroidetes*, *Firmicutes*, *α-Proteobacteria* and *β-Proteobacteria* decreased significantly in dams. After maternal OBS exposure, *Actinobacteria* increased in F_1_-20 d male mice, while *α-Proteobacteria* decreased; *Bacteroidetes* increased, and *Firmicutes* and *α-Proteobacteria* decreased in F_1_-20 d female mice. In F_1_-8 w mice, *Firmicutes* increased and *β-Proteobacteria* decreased in male, while *Bacteroidetes* and *β-Proteobacteria* decreased in female. High-throughput sequencing confirmed that sodium ρ-perfluorous nonenoxybenzene sulfonate significantly altered gut microbiota patterns in both dams and offspring. Biomarkers in dams and offspring varied after maternal OBS exposure, and differences were noticeable across genders and developmental stages. In dams, the abundance of *Desulfobacterota* and *Peptococcaceae* decreased, the abundance of *RF39* and *Lachnospiraceae* increased. Additionally, *Verrucomimicrobiota*, *Patescibacteria*, *Actinobacteriota*, and *Cyanobacteria* at the phylum level showed significant differences between dams and offspring, while *Verrucomimicrobiota* and *Patescibacteria* differed in male and female offspring. Furthermore, functional predictions indicated shifts in metabolic pathways in both generations after maternal OBS exposure. In a word, maternal OBS exposure disrupted gut microbiota and altered the metabolism processes in dams and offspring, offering insights into potential health risks associated with maternal OBS exposure.

## Introduction

The gut microbiota, known for its richness and diversity, encodes a vast array of genes impacting an organism’s development and overall health. For instance, they involve in maintaining intestinal integrity, metabolism equilibrium, immune function^1,2^. Its imbalance can lead to various metabolic disorders, such as type 2 diabetes, non-alcoholic liver disease and cardiometabolic disease^3^. Collectively, gut microbiota is closely related to our health.

Environmental pollution has become a major public health problem threatening human health. Numerous studies indicate that environmental pollutants can substantially alter gut microbiota compositions^4,5^urging the public to remain vigilant against these pollutants. Notably, pollutants like antibiotics, microplastics, endocrine disruptor, persistent organic pollutants (POPs) are the largest threat for gut microbiota^6,7^. Antibiotics, for example, can drastically reshape gut microbiota for impacting host health^8^. Broad-spectrum antibiotics like amoxicillin and cefotaxime, when administered during neonatal microbiota establishment, reduce *Bifidobacterium spp*. and increase *Klebsiella* and *Enterococcus spp*. in infants^9^. The latest report reveals that the total amount of microplastics deposits on the seafloor has tripled over the past 20 years and is a major pollutant in the ocean^10^. These microplastics have been found to significantly alter microbiota diversity in species like Zebrafish, Marine Medaka, and Chinese mitten crab^11–13^. Endocrine disruptors also significantly influence gut microbiota composition, leading to various pathological outcomes^14,15^. In the proposed new pollution control list, many environmental pollutants belong to POPs, known for their global health implications^16,17^. Perfluoroalkyl/polyfluoroalkyl substances (PFASs), a subset of POPs, can alter gut microbiota structure in organisms and produce biological toxicity^18–20^. Additionally, it also has certain generational toxicological effects^21^.

Sodium ρ-perfluorous nonenoxybenzene sulfonate (OBS), a type of PFASs, has garnered scholarly attention due to its toxicity^22^. The research showed that OBS has been detected in various environmental media, such as the human body, water, air. And the concentrations that are enriched in different media are different^23^. Recently, it was reported that OBS imbalanced of gut microbiota by affecting the villus height and the gene expression of intestines, as well as disrupting hepatic metabolism^24,25^. Additionally, OBS damaged the pancreatic health of mice by disturbing its gut microbiota^20^. Given the close connection between gut health and metabolic diseases, understanding the gut microbiota’s relationship with other bodily systems is critical for effective disease management.

Intergenerational impacts of environmental pollutants are also a critical concern in toxicology, with evidence showing that exposure to them can increase disease prevalence in subsequent generations without direct exposure^26,27^. Our previous study confirmed the genetic toxicity of OBS and it can accumulate in the serum and gut^28,29^. Here, we selected the concentration (0.5 mg/L and 5.0 mg/L) from earlier studies to examine if maternal OBS-induced gut microbiota changes are heritable. The potential toxicity of OBS was mainly assessed using 16 S rRNA sequencing analysis for providing the evidence to the safety assessment and rational use of OBS.

## Results

### Effects of maternal OBS exposure on the gut microbiota composition at the phylum level

**Fig. 1:**
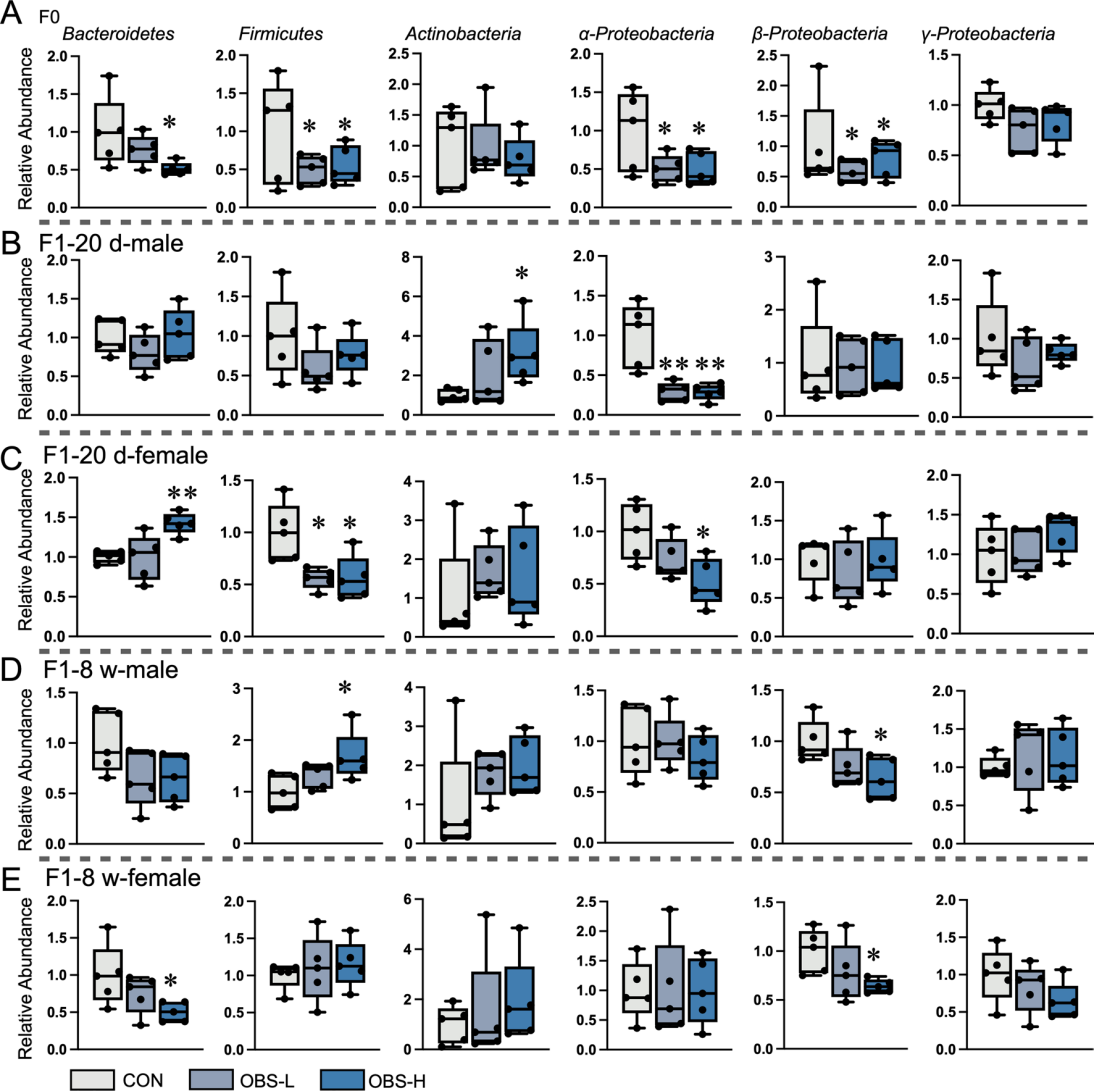
Effects of OBS on the composition of gut microbiota in mice. (**A**) Relative abundance of various gut microbiota in dams after maternal exposure. (**B**,**C**) Relative abundance of various gut microbiota in F_1_-20 d mice after maternal exposure. (**D**,**E**) Relative abundance of various gut microbiota in F_1_-8 w mice after maternal exposure. The presented values are the mean ± SEM (*n* = 5). Statistical significance was considered as *p* ≤ 0.05 with the asterisk compared with the control.

As shown in Fig. 1, at the phylum level, gut microbiota varied in F_0_ and F_1_ generations mice following maternal OBS exposure during pregnancy and lactation. After maternal OBS exposure, the relative abundance of *Bacteroidetes*, *Firmicutes*, *α-Proteobacteria* and *β-Proteobacteria* in dams significantly decreased with statistical significance (*p* < 0.05), while there was no significant change in *Actinobacteria* and *γ-Proteobacteria*. In offspring, gut microbiota differed from that of dam. *Actinobacteria* increased in F_1_-20 d male mice, while *α-Proteobacteria* decreased compared with the control, with statistical significance (*p* < 0.05). The relative abundance of *Bacteroidetes*, *Firmicutes*, *β-Proteobacteria* and *γ-Proteobacteria* did not exhibit significant changes (*p* > 0.05). In F_1_-20 d female mice, *Bacteroidetes* increased, and *Firmicutes* and *α-Proteobacteria* decreased compared with the control, with statistical significance (*p* < 0.05). The relative abundance of *Actinobacteria*,* β-Proteobacteria* and *γ-Proteobacteria* did not exhibit significant changes (*p* > 0.05). As the growth of mice, the changes in the gut microbiota persisted. Compared with the control, *Firmicutes* increased and *β-Proteobacteria* decreased in F_1_-8 w male mice with statistical significance (*p* < 0.05), the relative abundance of *Bacteroidetes*, *Actinobacteria*,* α-Proteobacteria* and *γ-Proteobacteria* did not exhibit significant changes (*p* > 0.05). While *Bacteroidetes* and *β-Proteobacteria* decreased compared with the control in F_1_-8 w female mice with statistical significance (*p* < 0.05), the relative abundance of *Firmicutes*, *Actinobacteria*,* α-Proteobacteria* and *γ-Proteobacteria* did not exhibit significant changes (*p* > 0.05).

### Effects of maternal OBS exposure on the structure of gut microbiota

**Fig. 2:**
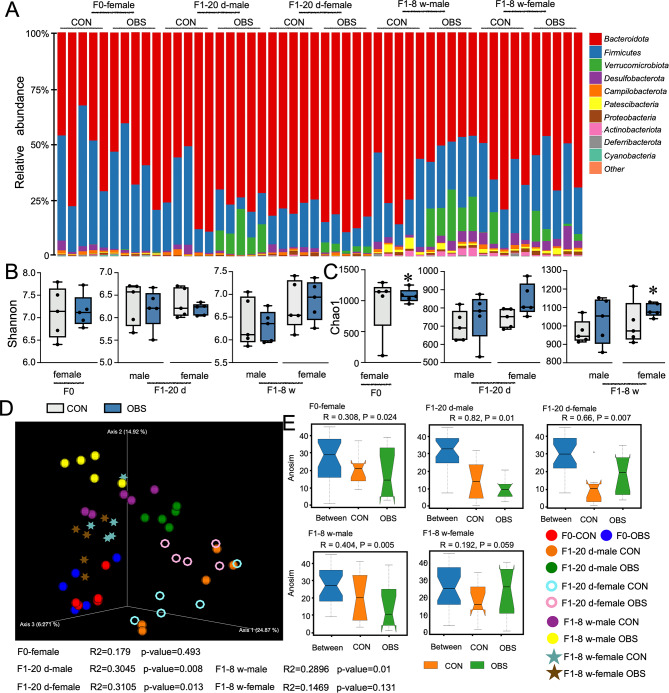
Effects of maternal OBS exposure on the structure of gut microbiota in F_0_ and F_1_ generations mice. (**A**) The microbiome composition profiles at the phylum level (each color represents one bacterial phylum). (**B**,**C**) The Shannon and Chao1estimates of the microbiota. (**D**) Bray-Curtis based PCoA estimates of microbiota. (**E**) Anosim analysis. The presented values are the means ± SEM (*n* = 5). Statistical significance was considered as *p* ≤ 0.05 with the asterisk compared with the control.

High-throughput sequencing showed that gut microbiota composition of gut microbiota in both dams and offspring after maternal OBS exposure corresponded to normal characteristics. The details of gut microbiota composition at the phylum level were in shown in Fig. 2A. The Fig. 2B and C showed that no significant difference in the Shannon index was observed between the groups without statistical significance (*p* > 0.05), the Chao1 index was upregulated in dams and F_1_-8 w female mice after maternal OBS exposure with statistical significance (*p* < 0.05). Moreover, results of β-diversity analysis showed that there were differences in the gut microbiota composition among dams, offspring mice at different life stages, and offspring mice of different genders, and differences also existed after OBS exposure (Fig. 2D). ANOSIM analysis (a non-parametric test) showed all groups 0 < R-value < 1, indicating intergroup differences exceeded intragroup differences and the grouping is significant. As shown in Fig. 2E, the ANOSIM analysis of β-diversity results showed that the groupings of F_0_ and F_1_ generations mice were reasonable.

### Differential species composition of gut microbiota after maternal OBS exposure

**Fig. 3:**
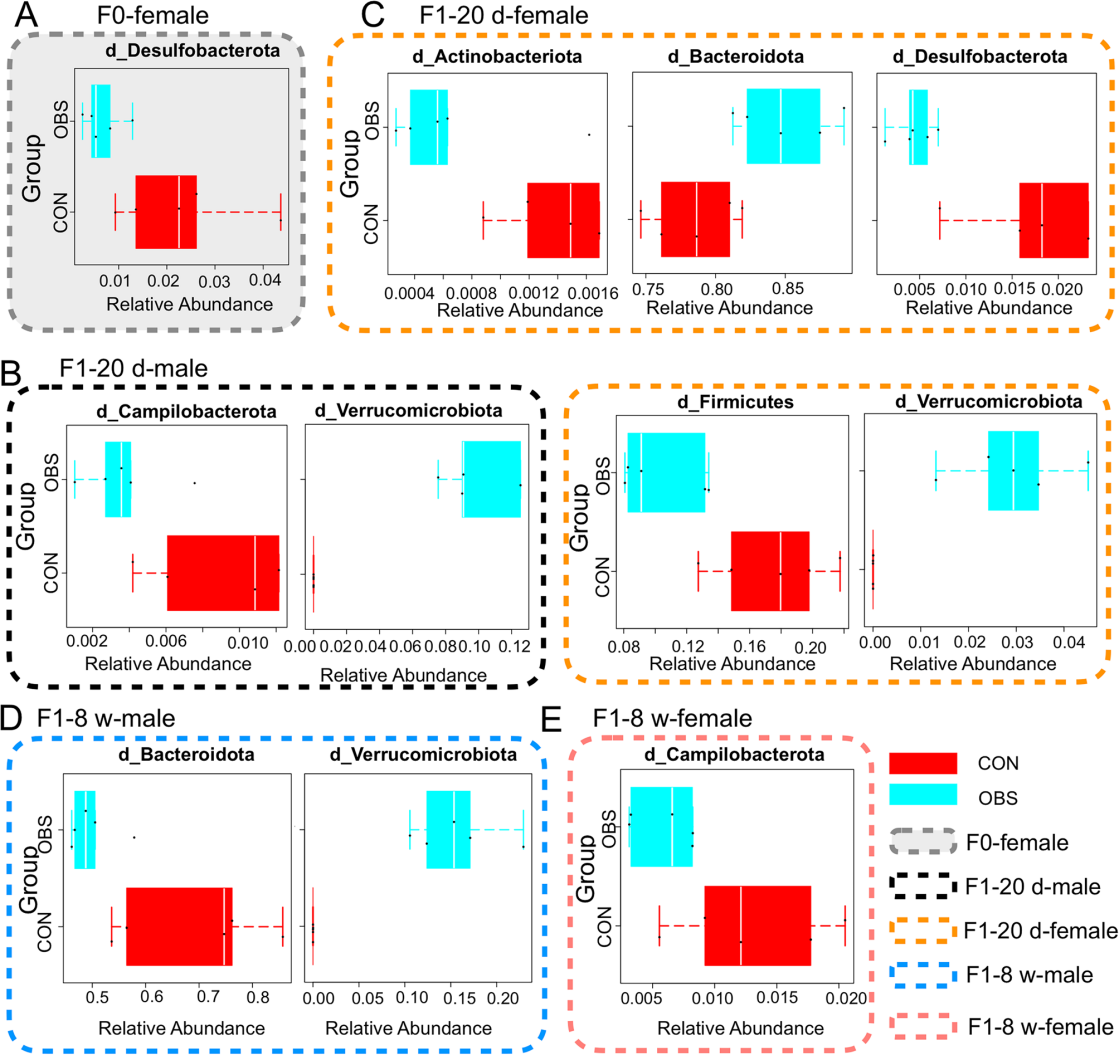
Differential species composition of gut microbiota in F_0_ and F_1_ generations mice. (**A**) Differential species in F0 generation. (**B**,**C**) Differential species in the male and female of F_1_-20 d mice. (**D**,**E**) Differential species in the male and female of F1-8 w mice.

Species differences were analyzed by one-way ANOVA, which is the Wilcoxon rank sum test of two groups. Results showed differences between control and OBS groups in gut microbiota of dams and offspring mice in Fig. 3. For dams, *Desulfobacterota* differed from the control (in Fig. 3A). In F_1_-20 d male mice, *Campilobacterota* and *Verrucomimicrobiota* differed (in Fig. 3B), while *Actinobacteriota*, *Bacteroidota*, *Desulfobacterota*, *Firmicutes*, *Verrucomimicrobiota* varied in F_1_-20 d female mice compared with the control (in Fig. 3C). With the growth and development of mice, new species differences arose in offspring mice. *Bacteroidota* and *Verrucomimicrobiota* differed from the control in F_1_-8 w male mice (in Fig. 3D), while *Campilobacterota* differed F_1_-8 w female mice (in Fig. 3E).

### Biomarkers of gut microbiota after maternal OBS exposure

**Fig. 4:**
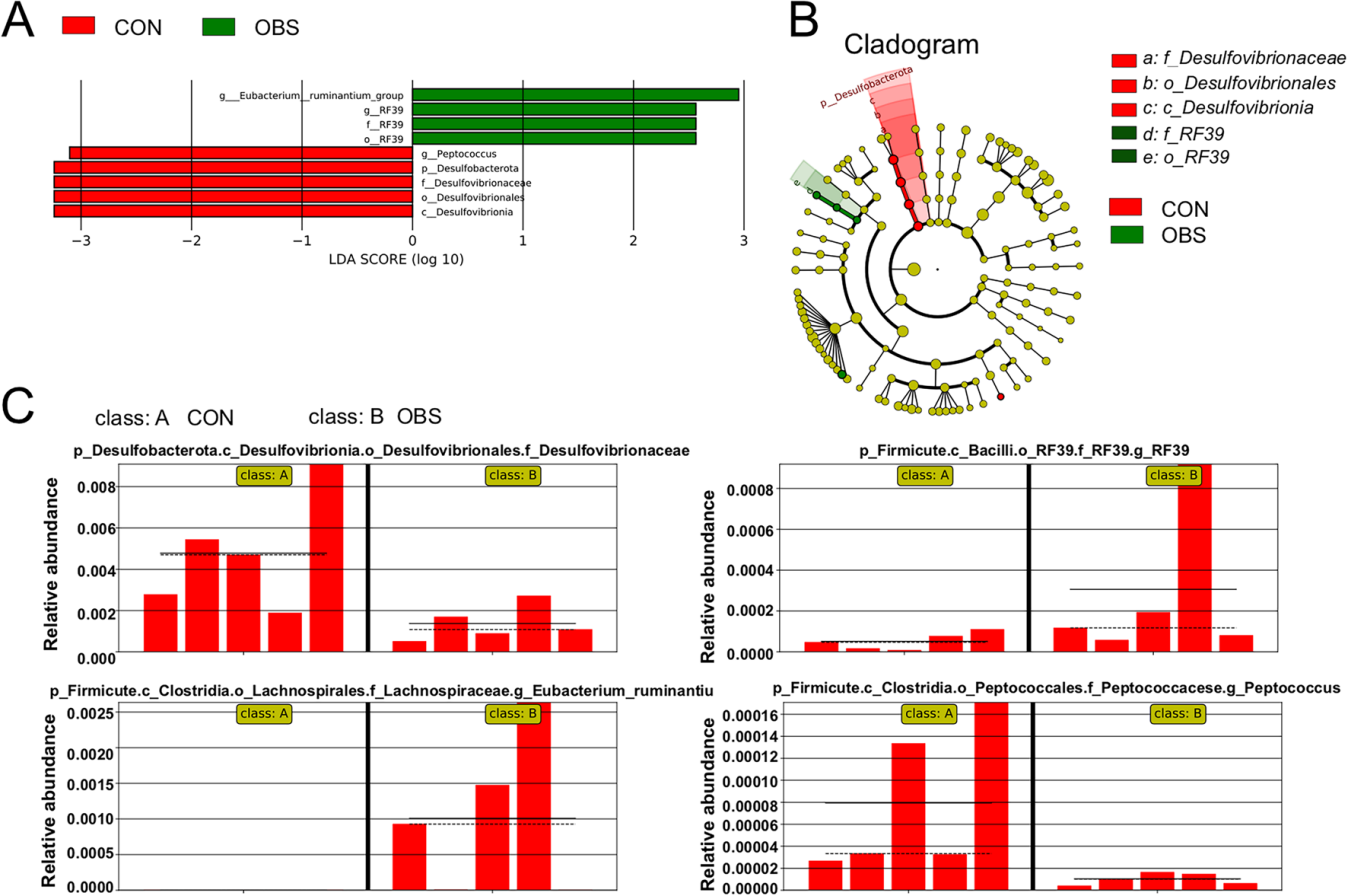
LEfSe analyses of gut microbiota in F0 generation mice after maternal OBS exposure. (**A**) The LDA scores calculated for characteristics at the OTU level. (**B**) LEfSe evolutionary branch diagram tree of species markers. (**C**) Relative abundances of *Desulfovibrionaceae*, *RF39*, *Lachnospiraceae* and *Peptococcaceae*.

**Fig. 5:**
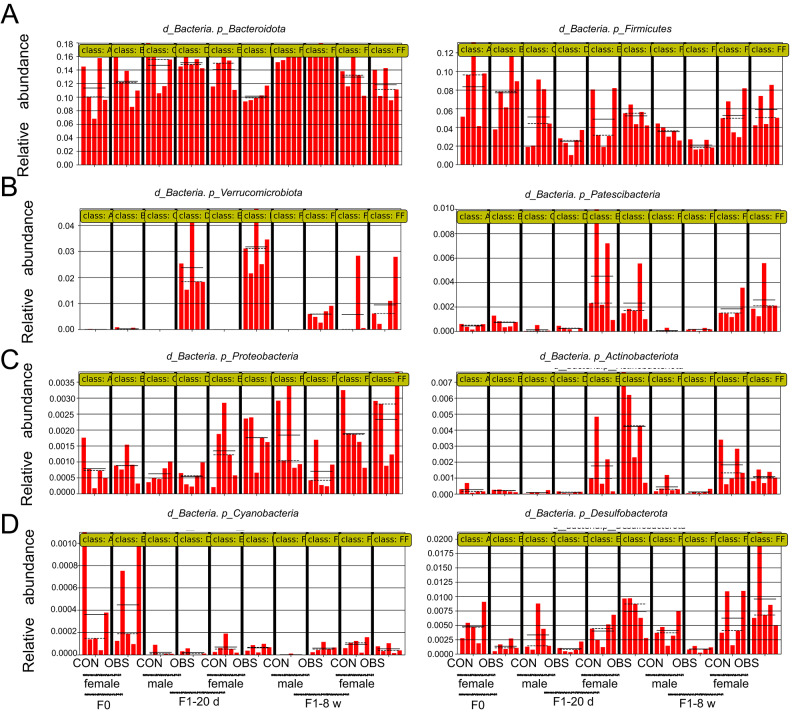
The abundance of biomarkers at the phylum level in F_0_ and F_1_ generations mice. (**A**) The abundance of *Bacteroidota* and *Firmicutes*. (**B**) The abundance of *Verrucomimicrobiota* and *Patescibacteria*. (**C**) The abundance of *Proteobacteria* and *Actinobacteria*. (**D**) The abundance of *Cyanobacteria* and *Desulfobacterota*.

LEfSe analysis (LDA Effect Size analysis) identified microbial biomarkers with significant intergroup variation after maternal OBS exposure. As shown in Figs. 4 and 5, Biomarkers differed by gender and age after maternal OBS exposure. In dams, the abundance of *Desulfobacterota*, within the Desulfovibrionaceae family, decreased after OBS exposure (in Fig. 4A-C). *Firmicutes* served as biomarkers in dams and their different families had vary trends, such as the abundance of *RF39* and *Lachnospiraceae* increased after OBS exposure, while the abundance of *Peptococcaceae* decreased (in Fig. 4C). Additionally, biomarkers (*Verrucomimicrobiota*, *Patescibacteria*, *Actinobacteriota*, and *Cyanobacteria*) at the phylum level showed significant differences between dams and offspring, while *Verrucomimicrobiota* and *Patescibacteria* differed in male and female offspring (in Fig. 5B).

### Metabolic pathways related to changes in gut microbiota after maternal OBS exposure

**Fig. 6:**
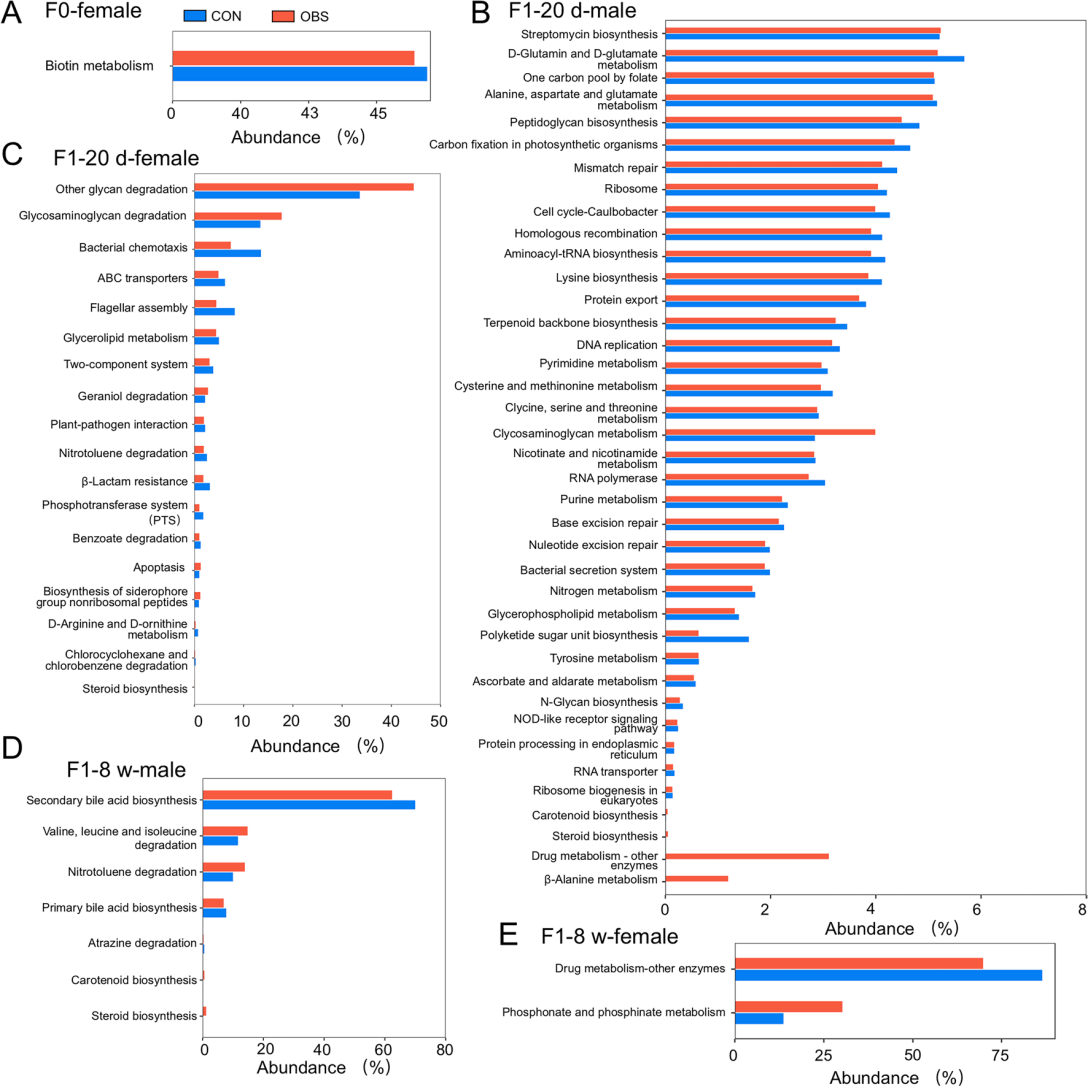
The enrichment pathway of differential microbiota in F0 generation mice after maternal OBS exposure. (**A**) in F_0_-female, (**B**) in F_1_-20 d-male, (**C**) in F_1_-20 d-female, (**D**) in F_1_-8 d-male, (**E**) in F_1_-8 d-female.

KEGG analyses indicated OBS-induced alterations in microbial communities were linked to varied metabolic pathways across different ages and genders. The Fig. 6A showed that changes were evident in biotin metabolism in F_0_ generation mice. As depicted in Fig. 6B–, pathways affected by maternal OBS exposure decreased as age increased in F_1_ generation. In F_1_-20 d male, 39 metabolic pathways (including Streptomycin biosynthesis, D-Glutamin and D-glutamate metabolism, Peptidoglycan bisosynthesis, Carbon fixation in photosynthetic organisms, et c) were maternal OBS exposure sites, while in F_1_-20 d females, 18 metabolic pathways (including Other glycan degradation, Glycosaminoglycan degradation, Bacterial chemotaxis, et c) were affected. For F_1_-8w adult males, 7 metabolic pathways (including Secondary bile acid biosynthesis, Valine, leucine and isoleucine degradation, Nitrotoluene degradation, Primary bile acid biosynthesis, Atrazine degradation, Carotenoid biosynthesis and Steroid biosynthesis) were involved, whereas for females, 2 metabolic pathways (Drug metabolism-other enzymes and Phosphonate and phosphinate metabolism) were altered.

## Discussion

Recently, OBS has been increasingly recognized as a risk factor for its potential health risks^22^. Maternal OBS exposure during pregnancy and lactation has been identified as a significant factor contributing to health issues in offspring. Here, pregnant C57BL/6 female mice received oral OBS through drinking water during pregnancy and lactation. The findings demonstrated notable shifts in gut microbiota composition at the phylum level in both dams and their offspring following maternal OBS exposure. This indicates that maternal OBS exposure adversely impacts the gut microbiota of offspring. Moreover, the high-throughput sequence of the 16 S rRNA gene’s V_4_ region indicated distinct differences in microbiota patterns between dams and offspring in both control and OBS-exposed groups. The dominant species in offspring mice varied by gender and developmental stages after maternal OBS exposure. Moreover, changes in offspring microbial communities induced by maternal OBS exposure were associated with alterations in various metabolic pathways across different ages and genders.

It is well established that gut microbiota holds vast amounts of information and plays a vital role in maintaining physiological processes^17,30,31^. The number of bacteria present within an organism is comparable to that of human cells^32^. This suggests that disruptions in gut microbiota could have significant health implications. In our study, maternal OBS exposure decreased the relative abundance of *Bacteroidetes*, *Firmicutes*, *Actinobacteria*, *α-Proteobacteria*,* β-Proteobacteria* and *γ-Proteobacteria* in dams, with varying effects observed in their offspring (Fig. 1). Among, Firmicutes are major bacteria in healthy organisms, typically in balance with Bacteroides^33,34^, but imbalances can occur^35^. It has been reported that gut microbiota dysbiosis may contribute to metabolic disorders^36–38^. And the abundance changes of some species at the *Bacteroidetes*, *Firmicutes* and *Proteobacteria* phylum levels were significantly associated with liver lipid degeneration in host metabolism^39^. Here, the pathways of steroid biosynthesis were altered in dam and offspring according to the KEGG functional prediction after maternal OBS-H exposure. This indicated that OBS can disturb the lipid metabolism by altering the gut microbiota. Additionally, it has been reported that maternal OBS exposure can cause early exposure in the offspring, which confirmed that the gut microbiota and metabolism of the offspring could unbalance due to maternal exposure^29^. High-throughput sequencing revealed differences between control and OBS-H exposure groups among various ages, including F_0_, F_1_-20d, and F_1_-8w mice, and ANOVA indicated species differences across these groups, and the difference species in dams was *Desulfobacterota*, F_1_-20 d male mice were *Campilobacterota* and *Verrucomimicrobiota*, F_1_-20 d female mice *were Actinobacteriota*, *Bacteroidota*, *Desulfobacterota*, *Firmicutes*, *Verrucomimicrobiota*. As mice matured, new differential species appeared in offspring exposed to OBS, such as *Bacteroidota* and *Verrucomimicrobiota* in F_1_-8w males, and *Campilobacterota* in F_1_-8w females. Additionally, the different metabolic pathways were altered in dams and offspring (F_1_-20d and F_1_-8w mice). As depicted in the Fig. 6A, biotin metabolism disrupted in dams after maternal OBS-H exposure. Additionally, the F_1_ generation exhibited the different OBS biomarkers after maternal OBS-H exposure, leading to changes in different metabolic pathways. In F_1_-20 d-male mice, 39 metabolic pathways acted as the sites for maternal OBS exposure, 18 metabolic pathways acted as the sites for maternal OBS exposure in F_1_-20 d-female mice. For adult mice (F_1_-8 w), 7 metabolic pathways were the sites for maternal OBS exposure in male mice and 2 metabolic pathways were the sites for maternal OBS exposure in female mice. It is further confirmed that different gut microbiota played different roles and regulate different metabolic pathways.

We all know that the gut microbiota involved in protective, metabolic, and structural functions for the host. And the structure of the gut microbiota undergoes significant and regular dynamic changes with age, which are closely related to the host’s growth and development, maturation of physiological functions, and aging process^31^. Therefore, as growth and development proceed, after early-life OBS exposure, the microbiota structure in mice changes, and their corresponding physiological functions also alter. Moreover, the disturbances experienced at different life stages vary. Based on ANOVA results for microbiota differences after maternal OBS exposure and differences across life stages, we investigated altered metabolic pathways in mice after maternal OBS exposure by analyzing corresponding biomarkers. The Fig. 4C presented that *Desulfobacterota*,* RF39*,* Lachnospiraceae* and *Peptococcaceae* were the biomarkers of OBS exposure in dams. *RF39*,* Lachnospiraceae* and *Peptococcaceae* belong to the *Firmicutes*, which, alongside *Desulfobacterota*, holds dominance in gut microbiota after metabolic disturbances^40^. Firmicutes are major bacteria in healthy organisms, typically in balance with Bacteroides^33,34^but imbalances can occur^35^. *Desulfobacterota* is correlated with liver metabolism^41^. Moreover, offspring mice (F_1_-20 d (male, female), F_1_-8w (male, female )) also revealed the different structure of gut microbiota after martenal OBS exposure. *Verrucomimicrobiota* was obviously upregulated in F_1_-20 d mice after martenal OBS exposure. Apparently, it is unresponsive to OBS, with a substantial presence of *Patescibacteria* and *Actinobacteriota* in mice at the 8-week stage (Fig. 5). *Verrucomicrobiota* is closely associated with the integrity of the intestinal mucus layer and anti-inflammatory functions. Its changes may reflect whether the intestinal barrier function is impaired, thereby disrupting metabolic balance^42^. It is reported that the presence of *Patescibacteria* may trigger the development of cancer^43^. *Actinobacteria* is one of the four major phyla in the gut microbiota. It plays an important role in improving the digestive function, regulating blood glucose, reducing blood lipid levels, and enhancing immunity^43^. These observations confirm that varied gut microbiota regulate distinct metabolic pathways, suggesting potential bacterial therapies for metabolic diseases based on specific microbiota functions and providing a new therapeutic direction for the treatment of metabolic diseases^44^.

This study also has certain limitations. First, the spontaneous behavior of dams during the exposure phase was not considered, so it is impossible to clearly distinguish the impact of perinatal OBS exposure on offspring. Second, no separate groups for prenatal exposure and lactational exposure were set up to fully explore the effects of indirect exposure at different periods on offspring. In future studies, targeted improvements can be made, with full consideration of the impacts of different factors.

## Conclusions

This study revealed that maternal OBS exposure during pregnancy and lactation primarily altered gut microbiota in dams and their offspring. The composition of gut microbiota in F_0_ and F_1_ generations was impacted with notable shifts in microbiota patterns, dominant species, biomarker and metabolic pathways in both generations. The effects of maternal OBS exposure were evident in both male and female offspring. The above findings underscore the importance of evaluating health risks associated with maternal exposure in future studies.

## Materials and methods

### Animals feeding

All animal experiment studies abided by the protocol approved by the Ethics Committee of Chinese Center for Changzhi Medical College, the Institutional Guidelines on Animal Experimentation at Changzhi Medical College (Approval number: DW2024051). All the experimental mice were purchased in the China National Laboratory Animal Resource Center (Shanghai, China). They were housed under controlled conditions at Changzhi Medical College until dissection. Throughout the study, all mice were kept individually in an animal facility maintained at 22 ± 1 °C with 12-hour light/dark cycle.

### OBS administration in mice

Based on the previous maternal toxicity study in mice, maternal OBS exposure has been shown to disrupt intestinal barrier and metabolism in dams and offspring^27,28^. Consequently, OBS concentrations of 0.0 (Control, CON), 0.5 (OBS-Low, OBS-L) and 5.0 (OBS-High, OBS-H) mg/L were selected to investigate effects on the gut microbiota. Detailed information about OBS can be found in prior research^29^. After confirmation of copulation via vaginal plug detection, female mice (dam, F_0_ generation) received the designated OBS doses in their drinking water until their offspring (F_1_ generation) were weaned. The F_1_ generation mice were weaned when they were 3 weeks old. While the basic diet was maintained, water was changed daily. Dam were sacrificed after weaning, their offspring were dissected at the age of 20 days (F_1_-20 d) and 8 weeks (F_1_-8 w). Before dissection, mice were anaesthetized with isoflurane to ensure humane treatment.

### DNA extraction and RT-qPCR amplification

Following the protocol by Wang et al.^25^DNA was extracted from the colon content using a commercial magnetic bead DNA isolation kit. Subsequently, portions of gDNA were amplified with the bacterial specific primers by Real-Time qPCR^45^. The cycling conditions for Real-Time qPCR and the primer sequences of the gut microbiota were conducted based on the studies by Engevik et al.^46^ and Wan et al.^35^.

### 16 S rRNA sequencing and analysis

The microbial genomic DNA extraction, variable 4 (V_4_) region amplification, and sequencing were performed as described in previous studies^47^. Analyses included alpha and beta diversity, anosim tests, inter group species differences, and functional metabolism predictions of various bacterial genera based on the data collected.

### Data analysis

Values are presented as the mean ± standard error of the mean (SEM). Statistical significance was considered as *p* ≤ 0.05 (*p ≤ 0.05, **p ≤ 0.01, ***p ≤ 0.001). Data were analyzed using GraphPad Prism version 9 (GraphPad Software) and SPSS 13.0 (SPSS, Chicago, Illinois), employing one-way ANOVA followed by Dunnett’s test between the control group and the OBS-exposed group.

## Data Availability

The datasets used and analyzed during the current study are available from the corresponding author upon reasonable request. Also, the datasets generated during the current study are available in the [DRYAD] repository, [DOI: 10.5061/dryad.9cnp5hqw0].
